# MXene derivatives: synthesis and applications in energy convention and storage

**DOI:** 10.1039/d0ra10018h

**Published:** 2021-04-30

**Authors:** Jinyi Sui, Xifan Chen, Yang Li, Wenchao Peng, Fengbao Zhang, Xiaobin Fan

**Affiliations:** School of Chemical Engineering and Technology, Tianjin University Tianjin 300072 China xiaobinfan@tju.edu.cn; Institutes of Physical Science and Information Technology, Anhui University Hefei 230601 China

## Abstract

Transition metal carbides or nitrides (MXene) have shown promising applications in energy convention and storage (ECS), owing to their high conductivity and adjustable surface functional groups. In the past several years, many MXene derivatives with different structures have been successfully prepared and their impressive performance demonstrated in ECS. This review summarizes the progress in the synthesis of MXene and typical Ti_3_C_2_T_*x*_ MXene derivatives with different morphologies, including 0D quantum dots, 1D nanoribbons, 2D nanosheets and 3D nanoflowers. The mechanisms involved and their performance in photocatalysis, electrocatalysis and rechargeable batteries are also discussed. Furthermore, the challenges of MXene derivatives in ECS are also proposed.

## Abstract 2

MXene derivatives with different morphology and applications in energy convention and storage.

## Graphical abstract

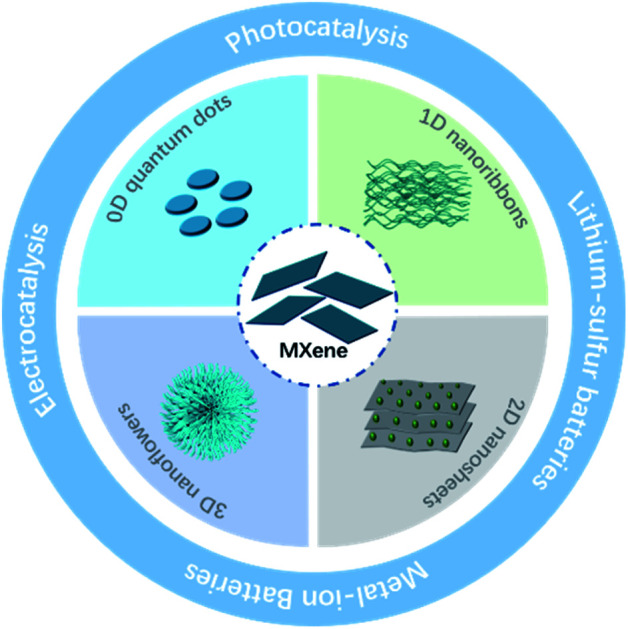

## 1 Introduction

Two-dimensional (2D) materials, including graphene, transition metal dichalcogenides,^1,2^ hexagonal boron nitrides,^3,4^ black phosphorene^5,6^ and silene^7,8^ have shown promising applications in energy convention and storage (ECS). Transition metal carbides or nitrides, known as MXenes, are the new-generation 2D materials first prepared in 2011.^9^ Typically, MXenes are prepared by the selective removal of a layer from MAX precursors (in which, M represents early transition metal element, A represents the group IIIA, IVA, X represents C and/or N).^9–11^ Until now, over 30 kinds of MXenes have been synthesized from the corresponding MAX phase. They have many fascinating properties for ECS applications: (1) large planes for charge storage and 2D channels for ion transfer resemble other 2D materials, (2) excellent electrical conductivity owing to the metallic bone, whose bandgap can be tuned by surface functional groups,^12^ and (3) abundant functional groups on the surface (–F groups, –OH groups, –O groups), resulting in the hydrophilic surface and their superior performance in the ECS application.^13,14^

**Scheme 1:**
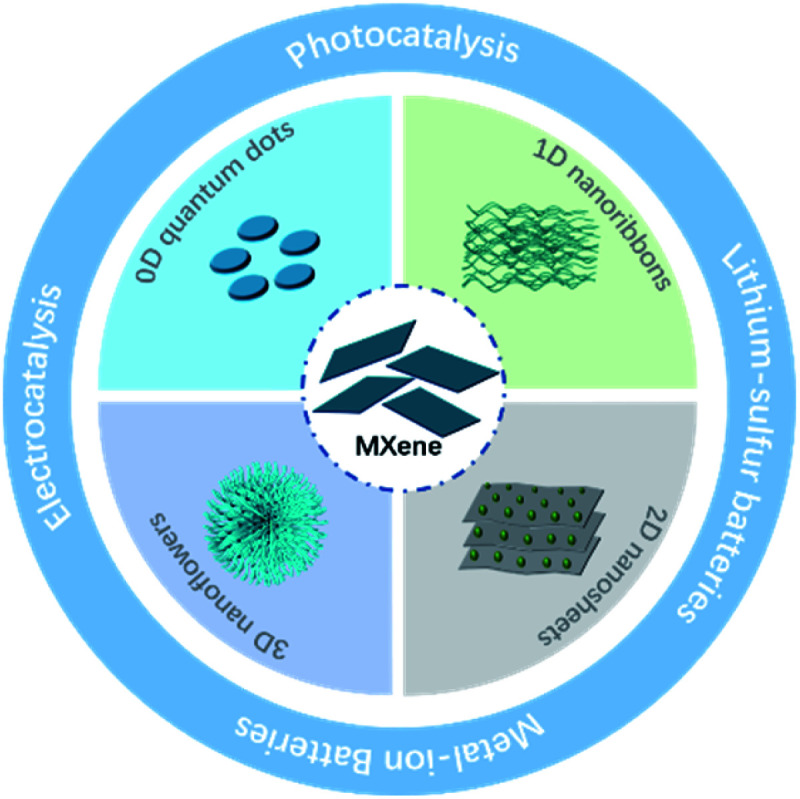
MXene derivatives with different morphology and applications in ECS.

In the past several years, many MXene derivatives with different structures, including 0D quantum dots, 1D nanoribbons, 2D nanosheets and 3D nanoflowers, have been successfully prepared. Their performance in ECS was also evaluated. However, the synthesis and application of these new MXene derivatives have never been comprehensively discussed. This review summarizes the recent progress in the synthesis of MXenes and their derivatives, as well as their performance in electrocatalysis, photocatalysis and rechargeable batteries (Scheme 1). Finally, the challenges and prospects of MXene derivatives in ECS are also proposed.

## 2 Synthesis of MXene

**Fig. 1:**
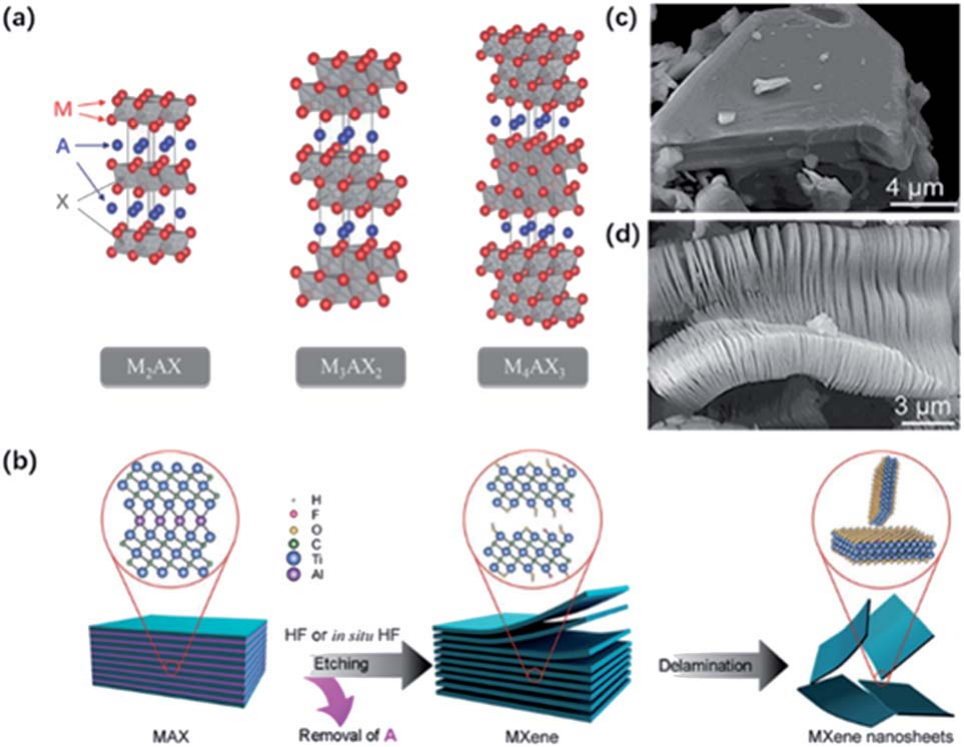
(a) Typical structure of MAX phases.^12^ This figure has been reproduced from ref. 12 with permission from American Chemical Society, copyright 2014. (b) Normal etching and delamination process of MXene.^21^ This figure has been reproduced from ref. 21 with permission from Elsevier, copyright 2019. (c, d) SEM graph for unreacted Ti_3_AlC_2_ particles (c) and HF-etched Ti_3_C_2_T_*x*_ (d).^22^ This figure has been reproduced from ref. 22 with permission from American Chemical Society, copyright 2012.

As shown in Fig. 1a, most MXenes are prepared from the MAX phase, where M and X atoms occupy the apex and center of the hexagonal crystal and A atoms interleave in MX layers. By taking advantage of this “laminar” structure, MX layers could be preserved after removing A atoms from MAX phases. However, the strong interaction between layers makes mechanical exfoliation difficult to generate 2D MXenes in contrast with other layered materials like graphene, where the weak van der Waals forces help maintain the structure.^15^ Therefore, many strategies have been developed to prepare MXenes. Current methods of preparing 2D MXenes and the differences between different routes are summarized in Table 1.

**Table 1:** Current methods of preparing 2D MXenes and the differences between different routes

Synthesis methods	Applicable MXenes (published)	Advantages	Disadvantages
HF/*in situ* HF etching method	Suitable for most MXenes	Simple and generally suitable	Environmentally harmful and dangerous
Molten salt etching process	Especially suitable for the nitrides MXenes	Successfully etched the nitrides MXene	Hard to be completely etched
Alkali treatment	Ti_3_C_2_T_*x*_	Environmentally friendly and more beneficial functional groups	Sensitive to the reaction conditions
Electrochemical etching	Ti_3_C_2_T_*x*_	Mild and large-scale flakes	Easy to over-etching

### 2.1 HF/*in situ* HF etching method

HF and *in situ* HF-etching are the main approaches to prepare MXene (Fig. 1b). Gogotsi and co-workers first reported the loosely stacked accordion-like MXene structure by selective etching of Al from bulk Ti_3_AlC_2_ in HF solution (50 wt%) (Fig. 1c and d).^9^ HF dissolved the Al layers by breaking Ti–Al bonds and released plenty of H_2_. Thereby, a violent bubbling phenomenon could be observed at the beginning of the reaction. At the same time, abundant functional groups including –F groups, –OH groups and –O groups were attached to Ti atoms on the surface, resulting in high hydrophilicity and unique electrochemical characters. Subsequently, Ti_2_CT_*x*_,^16^ V_2_CT_*x*_,^17^ Mo_2_CT_*x*_^18^ were successfully exfoliated by HF etching. It should be noted that the etching conditions depend on the structural properties of the MAX phase. For example, Nb_2_AlC required 90 h of etching time in 50 wt% HF, while Ti_2_CT_*x*_ only needed treatment of 10 h in 40 wt% HF. The result was also confirmed by theoretical calculations. The calculation results verified the essential longer time and higher HF solution concentrations when etching Al from Nb_2_AlC than from Ti_2_AlC, for the bond energy of Nb–Al (1.21 eV) is a little bit higher than that of Ti–Al (0.98 eV).^19^ However, it's worth mentioning that too tough an etching procedure will result in more defects on the surface, which might potentially influence the properties of MXene sheets.^20^ The successful delamination of Al layers greatly weakens the interaction between layers, making it easy to exfoliate MXene layers from the adjacent ones.

Considering the high risk and strong toxicity of HF, many efforts have been made to find milder and safer synthesis methods. In 2014, a few-layer MXene with larger interlayer spacing and fewer surface defects were achieved by using a mixture of LiF and HCl instead of HF solution.^23^ During the etching process, H^+^ and F^−^ were released to form HF *in situ*. The intercalated metal ions and water molecules took place of Al atoms, promoting the expansion of the interlayered space and weakening the interlayered interaction. Importantly, this modified process enabled the size and quality of sheets regulatable by controlling the concentration of LiF and HCl.^24^ The combinations of other fluoride salts (such as KF, NaF, and CaF_2_) and acid (H_2_SO_4_) are also used to replace the original etchant. However, the use of these fluorinated salts cannot completely avoid the formation of HF, and –F groups also limit the application of MXene in electrochemical fields.^9,25^

Moreover, many organic molecules have been applied as the intercalation agent to generate a few-layer MXene from multi-layer MXene, such as dimethyl sulphoxide (DMSO), and tetramethylammonium hydroxide (TMAOH). On account of the presence of these intercalants, the loosely stacking multi-layer MXene can be delaminated into few-layer sheets, even a monolayer with followed ultrasound or shaking treatment.^26,27^

### 2.2 Molten salt etching process

HF and *in situ* HF etching are efficient ways of producing carbides MXene while behaving badly in the etching of the nitrides MXene. Two possible reasons were proposed to demonstrate the difficulty in producing the nitrides MXene. First, the calculation results showed the cohesive energy of Ti_*n*+1_N_*n*_ was less than that of Ti_*n*+1_C_*n*_, which implied lower stability of Ti_*n*+1_N_*n*_. The formation energy of Ti_*n*+1_N_*n*_ is also higher than that of Ti_*n*+1_C_*n*_, which indicated that Al atoms were strongly bonded in the Ti_*n*+1_AlN_*n*_ structure.^28^ To overcome such problems, Gogotsi and co-workers adopted a mixture of molten salt as the etchant to obtain nitrides MXene.^29^ In particular, Ti_4_AlN_3_ was first mixed with molten salt (59 wt% KF, 29 wt% LiF, 12 wt% NaF) in a 1 : 1 mass ratio, and heated at 550 °C for 30 min under Ar atmosphere. Then, extra washing treatment with H_2_SO_4_ and DI water was required to dissolve the Al-containing fluorides. As confirmed from the XRD results, a strong broad (002) peak of Ti_4_N_3_T_*x*_ showed a shift from 7.6° to 6.3°, indicating the expanded interlayer distance. Also, no Al atom was observed according to EDX results, demonstrating the successful etching of nitrides MXene with molten salt.

### 2.3 Fluorine-free method

**Fig. 2:**
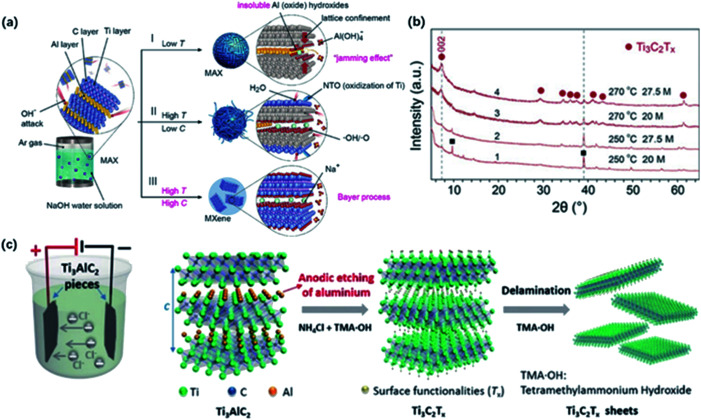
(a) The reaction of Ti_3_AlC_2_ with NaOH aqueous solution under different conditions. (b) XRD results of MXene treated in different temperatures and alkali concentrations.^30^ This figure has been reproduced from ref. 30 with permission from Wiley-VCH, copyright 2018. (c) The configuration of electrochemical cell and schematic diagram of the electrochemical etching of Ti_3_C_2_T_*x*_ with the combination of NH_4_Cl and TMAOH.^32^ This figure has been reproduced from ref. 32 with permission from Wiley-VCH, copyright 2018.

Recently, Li and co-workers reported a fluorine-free method to get the high-purity multilayer MXene *via* alkali hydrothermal treatment.^30^ High temperature and high NaOH concentration allow the complete dissolution of Al(oxide) hydroxides (Fig. 2a and b), and further delamination with TMAOH or DMSO and ultrasound treatment can help produce a few-layer MXene with smaller sizes.^31^ Different from fluorine-assisted methods, more O-containing groups are distributed on the surface instead of –F groups, which may be beneficial for the electrochemical performance. Although alkali treatment avoids the generation of HF, tough conditions may destroy the internal structure and create more defects on the surface.

Beyond this traditional wet chemical etching, electrochemical etching is an alternative way of preparing MXene sheets. Yang and co-workers developed an electrochemical etching method in a mixture of NH_4_Cl and TMAOH organic systems.^32^ Two pieces of bulk were used as the working electrode and counter electrode, while only the working electrode underwent the etching process (Fig. 2c).

With a constant applied potential (5 V), chloride ions worked as the Ti–Al bonds breaker and the generated NH_4_OH helped expand the edge. The exfoliated sheets were mostly single or double layers with an average lateral size of over 2 μm, larger than the traditional HF-etched MXene. In addition, Ti_3_C_2_T_*x*_ sheets achieved by electrochemical etching generally show a similar stacked morphology as bulk Ti_3_AlC_2_ without an obvious expansion because the reaction process does not involve violent gas release. It should be noted that the selection of electrolytes also plays a crucial role in the result of etched MXene. For example, electrochemical treatment in NaCl, HCl solutions always generates amorphous carbon, blocking further etching process.^33^ Sun and co-workers found that a three-layer structure composite was generated when dealing MAX with 2 M HCl aqueous electrolyte.^34^ This hybrid consisted of carbon-derived carbides (CDCs), unetched MAX and MXene, which needed further purification to harvest pure MXene sheets.

Above all, by utilizing the intensity difference between M–A bonds and M–X bonds, MXene could be obtained from bulk MAX phases through specific etching methods, such as HF acid etching, molten salts etching and other fluorine-free routes. It should be noted that the etching conditions varied widely with the M–A bond energies.^19^ The higher the M–A bond energies, the longer etching time and higher etchant concentration are required. Furthermore, MAX particles' size plays an important role in the etching rate and reaction conditions.^17^ Pre-treating the MAX powder by attrition milling could greatly reduce the etching time without enhancing overall yield.

### 2.4 Other bottom-up method

Except for the etching method mentioned above, other bottom-up methods, such as chemical vapor deposition,^35,36^ template method,^37,38^ plasma-enhanced pulsed laser deposition (PEPLD),^39^ have been created to fabricate ultrathin 2D MXene with good crystallinity, especially for Mo_2_C materials. Gogotsi and co-workers synthesized α-Mo_2_C crystals on the molybdenum–copper foil under the CH_4_ atmosphere, where the Mo atoms react with C atoms from CH_4_ at the melt surface.^35^ The obtained Mo_2_C exhibited an ordered crystal structure of tungsten carbide crystal according to the HRTEM image. These methods could better tune the size and thickness of 2D MXene crystals, enriching synthesis approaches to 2D MXene sheets.

## 3 Synthesis of Ti_3_C_2_T_*x*_ MXene derivatives

MXenes have shown great potential in ECS because of the 2D lamellar structure, high conductivity and abundant functional groups on the surface. Recently, the design of MXene derivatives with different morphologies and properties has drawn increasing attention, which shows improved performance in ECS compared to pristine MXene. The morphology engineering could be realized by choosing different synthesis methods. In this section, we summarize the synthesis methods of typical Ti_3_C_2_T_*x*_ MXene derivatives, including 0D quantum dots (0D QDs), 1D nanoribbons (1D NRs), 2D nanosheets (2D NSs), and 3D nanoflowers (3D NFs). Specific synthetic routes to Ti_3_C_2_T_*x*_ MXene derivatives of different morphologies and their further application are listed in Table 2.

**Table 2:** Summary of synthetic route of MXene derivatives with different morphology

Sample	Structure	Synthetic method	Application	Ref.
Ti_3_C_2_ QD	0D QDs	Hydrothermal	Multicolor cellular imaging	40
Ti_3_C_2_ QD	0D QDs	Hydrothermal	Li–S battery	41
Ti_3_C_2_ QD/g-C_3_N_4_	0D QDs	Hydrothermal	Photocatalytic hydrogen production	42
Ti_3_C_2_ QD	0D QDs	Solvothermal	—	43
N-Ti_3_C_2_ QD	0D QDs	DETA-assisted solvothermal	Selective Cu^2+^ detection	44
Ti_3_C_2_ QD	0D QDs	Ultrasound	Fe^3+^ detection	45
Ti_3_C_2_ QD	0D QDs	Reflux	—	46
Ti_3_C_2_ NR	1D NRs	Alkali treatment of MXene	Na/K-ion battery	47
Ti_3_C_2_ NR	1D NRs	Alkali treatment of MXene	Li–S battery	48
Na_0.23_TiO_2_/Ti_3_C_2_	1D NRs	Partial alkali treatment	Li/Na-ion battery	49
Ti_3_C_2_ NR/Ti_3_C_2_	1D NRs	Partial alkali treatment	Humidity sensor	50
h-Ti_3_C_2_/CNTs	1D NRs	Alkali treatment of MXene	Na–O_2_ batteries	137
Ti_3_C_2_ NR	1D NRs	Alkali treatment of MAX followed by HF etching	HER	51
TiO_2_/Ti_3_C_2_	2D NSs	Hydrothermal	Hexavalent chromium removal	52
TiO_2_/C	2D NSs	Hydrothermal and annealing	Photocatalytic water splitting	53
Ti_3_C_2_/TiO_2_/PANI	2D NSs	Hydrothermal	Electromagnetic wave absorption	134
PANI@TiO_2_/Ti_3_C_2_T_*x*_	2D NSs	Hydrothermal	Supercapacitors	138
TiO_2_/Ti_3_C_2_	2D NSs	Solvothermal		54
TiO_2_/C	2D NSs	Calcination in CO_2_ atmosphere	Photocatalytic water splitting	53
N-TiO_2_/C	2D NSs	Calcination of modified MXene in CO_2_ atmosphere	Photodegradation	55
TiN/C	2D NSs	Calcination in N_2_ atmosphere	Supercapacitor	56
NTO/KTO	3D NFs	Hydrothermal in alkali solution	Na/K-ion battery	57
Ti_3_C_2_–TiO_2_	3D NFs	Alkali treatment followed by ion change and heating	Photocatalytic water splitting	58
TiO_2_–Ti_3_C_2_–	3D NFs	Hydrothermal in alkali solution	Photocatalytic water splitting	133
Na_2_Ti_3_O_7_@C	3D NFs	Hydrothermal in alkali solution	Na-ion battery	135
HNTO/CS	3D NFs	Hydrothermal in alkali solution	K-ion battery	136
TiO_2_/C	3D NFs	Alcohol-thermal decomposition	Dehydrogenation of sodium alanates	59

### 3.1 Synthesis of 0D quantum dots

The 0D quantum dots are a kind of common 2D material derivatives with size advantage and unique optical properties originating from the quantum confinement and edge effects. In the past decades, quantum dots derived from different 2D materials, such as graphene and MoS_2_, are successfully produced.^60,61^ The MXene quantum dots (MQDs) were first prepared by Xue and co-workers in 2017.^40^ The as-prepared MQDs showed the same hexagonal lattices as the MAX phase, while exhibited smaller diameters of less than 10 nm.^62^

**Fig. 3:**
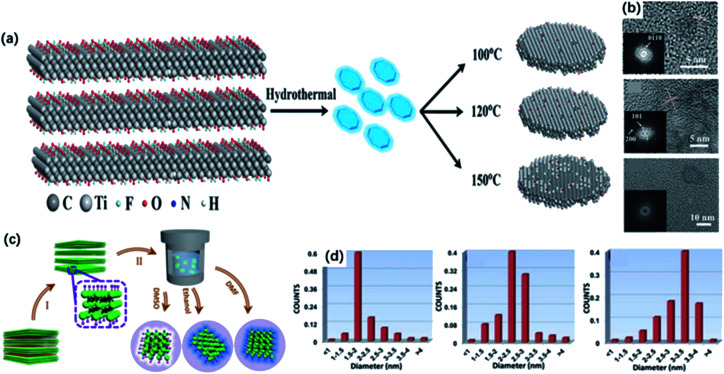
(a) Scheme for the synthesis of Ti_3_C_2_ QDs by a hydrothermal method at different temperatures. (b) HRTEM graphs of MQDs treated in 100 °C, 120 °C, 150 °C, respectively.^40^ These figures have been reproduced from ref. 40 with permission from Wiley-VCH, copyright 2017. (c) Schematic diagram of the preparation of MQDs *via* solvothermal treatment in different solvents, including DMF, DMSO and ethanol. (d) Size distributions of DMF-treated, DMSO-treated, ethanol-treated MQDs.^43^ This figure has been reproduced from ref. 43 with permission from Wiley-VCH, copyright 2018.

Generally, 0D MQDs are exfoliated from selected precursors *via* chemical or physical methods, such as hydrothermal/solvothermal,^63,64^ ultrasound treatment^65,66^ and reflux.^46^ Defects are created in this process and are served as the cutting points so that the layer sheets could be cut into smaller QDs. Up to now, the hydrothermal method has been considered the most common way of preparing MQDs. The resulting MQDs exhibit good solubility in water and ethanol. Xue and co-workers synthesized the monolayer MQDs with the quantum yields of 10% using the hydrothermal method (Fig. 3a).^40^ The average lateral particle sizes were tuned from 2.9 nm to 6.2 nm when the temperature rose from 100 to 150 °C. To be noted, the crystal structure also changed with the reaction temperature. In particular, MQDs treated at 100 °C showed the same structure of MXene, as confirmed by the lattice spacing (0.266 nm) assigned to the (0110) facet of MXene. When the temperature got higher, the (101) facet of TiO_2_ could be observed at 120 °C, and higher temperature (150 °C) led to the violent etching of Ti atoms and the undesirable formation of amorphous carbon dots (Fig. 3b).

Except for the hydrothermal process, solvothermal is another effective way to prepare MQDs by changing the reaction medium, which determines the sizes and quantum yields of MQDs. Xu and co-workers studied the relevance between solvents and properties of MQD *via* the solvothermal treatment of MXene.^43^ As shown in Fig. 3c, MQDs were prepared at 120 °C for 6 h in ethanol, dimethylformamide (DMF) and dimethyl sulfoxide (DMSO). As a result, MQDs treated in DMF possessed the largest average diameters of 3.3 ± 0.2 nm and the highest quantum yields of 10.7% among them, whereas the quantum yields of MQDs treated with ethanol and DMSO are only 6.9% and 4.1%, respectively (Fig. 3d). It was suggested that the physical properties of solvents, such as combined action of polarity, oxidation and boiling points could exert influences on the sizes and optical properties of MQDs. Specifically, high solvent polarity led to a strong interaction between solvent molecules and MXene sheets, and the low boiling point of the solvent caused higher pressure during the reaction. Under such experimental conditions, both resulted in smaller sizes and higher yields of MQDs. In addition, heteroatom doping during the hydrothermal/solvothermal treatment could significantly improve the electronic properties of QDs, induce more active sites on the surface, as well as obtain higher yields.^67–69^

**Fig. 4:**
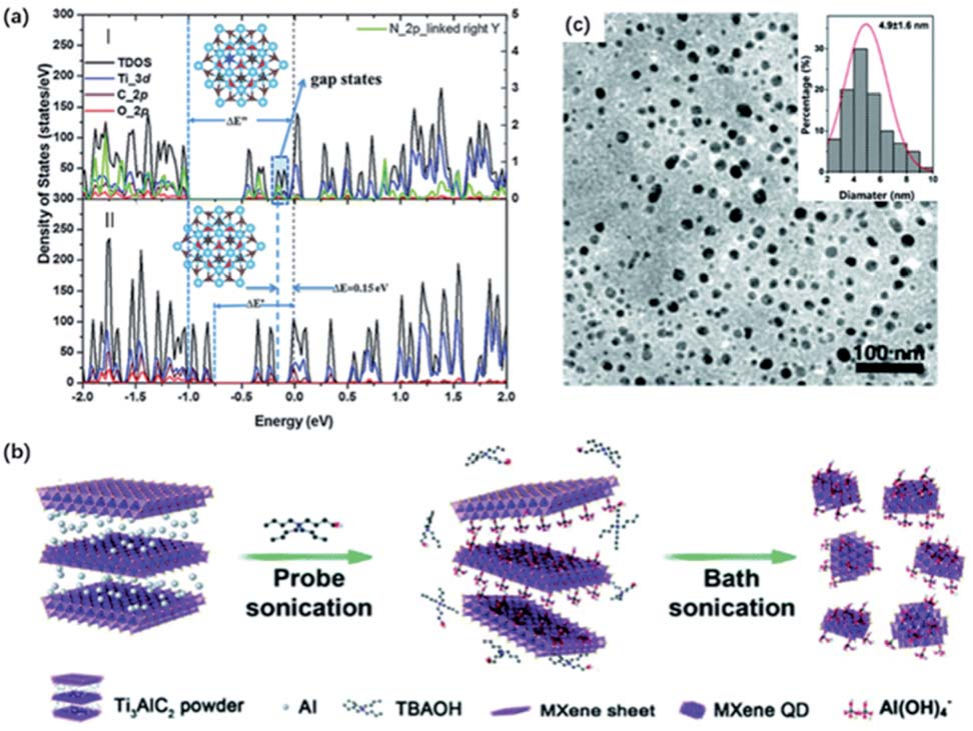
(a) Total and projected density of states by DFT calculation of (I) N-doped Ti_3_C_2_ QDs and (II) Ti_3_C2 QDs.^69^ This figure has been reproduced from ref. 69 with permission from Royal Society of Chemistry, copyright 2018. (b) Schematic diagram of the preparation of MXene QDs *via* sonication treatment. (c) TEM graph of the sonication treated MXene QDs. Inset (top-right) in (c): lateral sizes of MXene QDs by statistical analysis.^66^ This figure has been reproduced from ref. 66 with permission from Royal Society of Chemistry, copyright 2017.

Feng and co-workers designed a DETA-assisted solvothermal route to obtain *in situ* nitrogen-doped MQDs in DMF solution.^44^ N-MQDs showed smaller particle sizes than MQDs because of the contribution of N to the increased surface defects. The N-MQDs exhibited improved fluorescence emission traits, which probably resulted from the strong electron-donation effect. Xu and co-workers developed N-MQDs through a hydrothermal process using ethylenediamine as a nitrogen source, which showed the highest quantum yields (18.7%) among the past reports.^69^ The effect of N-doping on quantum yields was further verified by DFT calculations, where O-terminated Ti_3_C_2_ was used. In terms of the calculation results, a prominent gap state and wide energy gap could interpret the increased lifetime of carriers and improved quantum yields (Fig. 4a). Also, nitrogen doping introduced gap states close to the LUMO, that is, nitrogen doping of MQDs could accelerate the electron migration and eventually increase the carrier lifetime. Sometimes, nitrogen-containing solvents can serve as the nitrogen source without the additional agent as well. Lu and co-workers prepared highly fluorescent N-MQDs using dimethylformamide (DMF) as both, the solvent agent, and doping addictive, showing a significantly higher quantum yield of 11.13% than with ethanol (1.09%) and water (0.34%) system.^67^

Moreover, ultrasound treatment could be introduced to prepare MQDs, breaking MXene into small pieces and exposing more edges and sites.^70,71^ Zhang and co-workers designed a facile one-step ultrasound method, where delaminated MXene were sonicated for 10 h to obtain MQDs (Fig. 4b).^45^ The resulting MQDs exhibited spherical-like morphology and displayed monodisperse uniform distribution (Fig. 4c). Yu and co-workers combined TMAOH-assisted exfoliation and mechanical treatment to produce MQDs from pristine bulk Ti_3_AlC_2_ instead of using fluorine solvents.^66^ Pre-sonication of Ti_3_AlC_2_ was applied to expose more fresh edges and surfaces for the TBAOH etching process.

It is worth mentioning that the size of MQDs primarily depends on the temperature and reaction medium. Tuning the reaction conditions is important for achieving different sizes and higher yields of MQDs. The average lateral particle sizes become larger when the temperature rises, while the too high temperature may lead to the undesirable change of crystal structure. The solvent medium with a low boiling point also contributes to smaller sizes of MQDs because of the larger pressure during the reaction. In addition, heteroatom doping during reaction could decrease the average sizes of MQDs and achieve higher quantum yields, thanks to the prominent gap state and wide energy gap. More efforts could be made to design the expected structure by optimizing the reaction conditions.

### 3.2 Synthesis of 1D nanoribbons

One-dimensional nanomaterials, such as carbon nanotubes, are widely used in energy storage and wearable device applications owing to their high specific surface area, abundant exposed active sites and great mechanical reliability.^72–75^ Inspired by the special properties, nanoribbons derived from 2D materials like graphene were successfully synthesized by breaking the chemical bonds.^76^

**Fig. 5:**
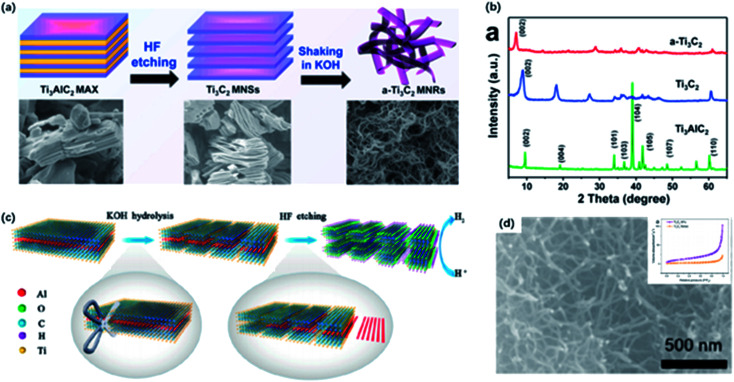
(a) Schematic of preparation of Ti_3_C_2_ MNRs. (b) XRD patterns of Ti_3_C_2_ MNRs, Ti_3_C_2_ and Ti_3_AlC_2_.^47^ This figure has been reproduced from ref. 47 with permission from Elsevier, copyright 2017. (c) Schematic showing the synthesis of Ti_3_C_2_ NF directly from Ti_3_AlC_2_*via* KOH-assisted treatment. (d) High magnification SEM image of Ti_3_C_2_ NFs. Inset (top-right) in (d): N_2_ adsorption–desorption isotherm of Ti_3_C_2_ NFs and Ti_3_C_2_ flakes.^51^ This figure has been reproduced from ref. 51 with permission from American Chemical Society, copyright 2017.

MXene nanoribbons (MNRs) were first fabricated by continuous shaking treatment of HF-etched Ti_3_C_2_ in aqueous 6 M KOH solution for 72 h at room temperature (Fig. 5a).^77^ Few MNRs appeared at the first hour, and longer MNRs were generated with the longer treatment time. The obtained MNRs displayed a narrow width of 6–22 nm. As confirmed from the XPS spectrum, the signal of F1s was almost unobservable after alkalization and a distinguishable signal of the Ti–O group appeared, indicating the transformation of –F groups to –OH groups. Also, the XRD pattern showed that the (002) peak of Ti_3_C_2_ was shifted to 7.1° from 8.9°, suggesting the expansion of interlayer spacing resulting from the intercalation of K^+^ into layers (Fig. 5b). Given little research has been done on MNRs, Lian and co-workers proposed a possible forming mechanism of MNRs. Initially, alkali treatment promoted the transformation of the surface group from –F to –OH, which strengthen the rapid adsorption and the intercalation of K^+^ into layers. Afterward, the mechanical shaking treatment enhanced the diffusion of OH^−^ and K^+^ along the channels of interlamination contributing to the O-terminated MNRs, and thus, splitting MNRs from the delaminated sheets. Li and co-workers treated MXene with different concentrations of KOH solutions (6, 12 and 24 mol L^−1^) for 2, 10 and 20 h to determine the key impacts on the morphology and quantity of MNRs, including the concentration of KOH solutions and the reaction time.^50^ As a result, it was demonstrated that the diameter and quantity of nanowires were proportional to the KOH concentration, while overlong reaction time might result in the agglomeration of nanoribbons. Yuan and co-workers developed a new strategy to fabricate MNRs directly from the MAX phase *via* the KOH-assisted treatment (Fig. 5c).^51^ Ti_3_AlC_2_ powders were first added to 6 M KOH and stirred for 96 h at room temperature, and MNRs with the width of 50 nm were harvested without being destroyed or collapsed after the HF-etching treatment (Fig. 5d). In the process, OH– served as a “scissor” to destroy Ti–C bonds in Ti_3_AlC_2_, fabricating the crack propagating to the edge. Interestingly, the pre-treatment with alkali solution can shorten ion transfer path and increase surface area, making the follow-up etching more efficient. Inspired by this study, a one-step alkalization treatment was developed to generate MNRs from Ti_3_AlC_2_ without using a fluorine-containing etchant.^78^ Sugarcane-like transitional etching products were formed by the hydrothermal treatment after grounding a different ratio of MAX and KOH powders with pretty small amounts of water added. With the increase of KOH content and longer reaction time, MNRs with narrow width were finally obtained.

### 3.3 Synthesis of 2D nanosheets

Ti_3_C_2_T_*x*_ sheets are promising candidates for ECS due to their specific surface area and high conductivity in a 2D planar structure. Whereas the oxidation stability of Ti_3_C_2_T_*x*_ is unsatisfactory due to the exposure of the large portion of Ti atoms on the surface.^79^ After exposing in the open air at room temperature, anatase TiO_2_ nanoparticles might form at the edge sites only in a week according to fast Fourier transform (FFT).^80^ However, the rich titanium atoms could also act as nucleating sites managing the *in situ* growth of TiO_2_ on Ti_3_C_2_ layers during oxidation in the meanwhile. Importantly, the unique properties of TiO_2_ and the interface between different components in the heterostructure improve the performance in ECS, particularly in photocatalysis compared to a single component. According to previous reports, hybrid nanosheet structure has been fabricated by partial or complete oxidation of MXene.^53,81,82^

**Fig. 6:**
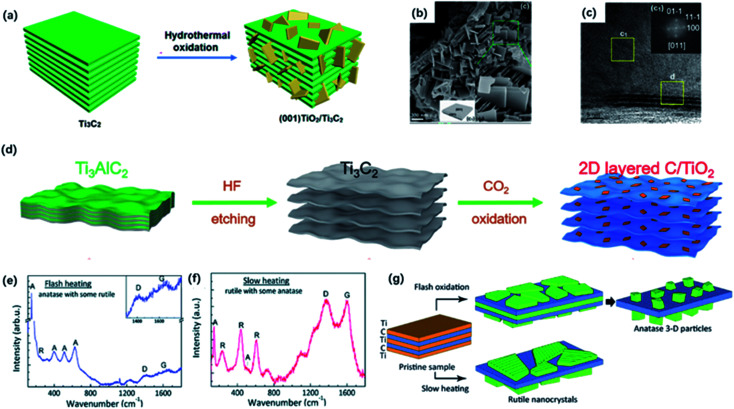
(a) Schematic of the preparation of (001) TiO_2_/Ti_3_C_2_ composite by hydrothermal treatment. (b) SEM photos of (001) TiO_2_/Ti_3_C_2_ composite. (c) TEM image of (001) TiO_2_/Ti_3_C_2_. Inset (top-right) in (c): the indexed FFT image of TiO_2_ showed the exposing (001) facets.^90^ This figure has been reproduced from ref. 90 with permission from American Chemical Society, copyright 2016. (d) Scheme diagram of the preparation of 2D-layered C/TiO_2_ hybrids *via* CO_2_ calcination.^82^ This figure has been reproduced from ref. 82 with permission from Wiley-VCH, copyright 2017. (e, f) Raman spectra of the final product of flash heating and slow heating.^97^ (g) Schematic diagram of two oxidation regimes including flash oxidation and slow heating. This figure has been reproduced from ref. 97 with permission from Royal Society of Chemistry, copyright 2014.

The oxidation degree of the obtained MXene hybrid depends on the condition of the synthesis method. The controlled oxidation of Ti_3_C_2_T_*x*_ MXene could be a promising method to prepare functional TiO_2_/MXene hybrids. It is reported that hydrothermal treatment is a common way to get partially oxidized MXene under relatively low temperatures.^83,84^ Zhang and co-workers constructed the hierarchical accordion-like TiO_2_/Ti_3_C_2_ hybrid *via* a facial hydrothermal strategy.^85^ TiO_2_ nanoparticles were uniformly covered on the MXene sheets, contributing to an expanded interlayer space. Importantly, the reaction solvent also determines the nanoparticle size of TiO_2_.^86,87^ As we know, the particle size influences the photocatalytic activity of TiO_2_.^132^ It has been reported that reducing the size of TiO_2_ particles could decrease the path of holes during the photocatalytic process, contributing to the increased photocatalytic efficiency. In addition, the smaller particles possessed a larger specific surface area, increasing the contact area of TiO_2_ and MXene.^54^ Zhang and co-workers figured out the existence of a moderate amount of ethanol under the hydrothermal condition could effectively reduce the size of TiO_2_ nanoparticles.^54^ Ethanol weakened the contact between the TiO_2_ nucleus and water because of the relatively high viscidity and molecular weight, leading to the formation of smaller-sized TiO_2_. In addition, hybrids increase the interlayer distance and improve the electrochemical properties.^88^ Moreover, it has been demonstrated that the exposed facet of TiO_2_ could be controlled by adding a morphology-directing agent.^89^ In order to design the favorable growth of TiO_2_, Peng and co-workers developed a new strategy of synthesizing the TiO_2_/Ti_3_C_2_ heterojunction nanocomposites in 1.0 M HCl solution *via* the hydrothermal treatment of Ti_3_C_2_ (Fig. 6a).^90^ With the addition of 0.1 M NaBF_4_, which served as the morphology-directing reagent, the exposing (001) facets of anatase TiO_2_ could be selectively controlled without any additional Ti sources (Fig. 6c). This modification method has been widely utilized to manage the favorable growth of TiO_2_ with specific exposing facets on Ti_3_C_2_ sheets.^84,91,92^ The *in situ* generated TiO_2_ nanoparticles also exhibited an aggregation distribution on the surface and interlayers, which contributed to the sandwich structure (Fig. 6b).^52,93^ Along with the increased reaction time, the size of nanoparticles gradually increased as well.^52^

Except for the hydrothermal process, calcination could be an alternative way of producing oxidized MXene. It has been shown that the heat treatment of Ti_3_C_2_T_*x*_ in the air or flowing CO_2_ atmosphere could result in complete oxidation of TiO_2_ embedded in the amorphous carbon layer by controlling the appropriate temperature.^94,95^ In the early time, Naguib and co-workers first studied the fast flash oxidation from Ti_3_C_2_T_*x*_ to TiO_2_@C composites at 1150 °C for 30 seconds under air atmosphere.^96^ However, it seemed hard to control the reaction during the violent oxidation process. Gogotsi and co-workers explored two oxidation regimes including flash oxidation and slow heating process to confirm a different mechanism of oxidation (Fig. 6d).^97^ During flash oxidation, the Ti atoms on the surface were first oxidized into planar anatase thin film (Fig. 6e). With more innermost Ti atoms migrating to the surface, vertical nanoparticles were generated on the nanosheets. By contrast, the slow heating process only led to the formation of the thin rutile TiO_2_ sheet on the surface (Fig. 6f). Moreover, Yuan and co-workers reported 2D layered TiO_2_/C hybrid *via* one-step CO_2_ oxidation of Ti_3_C_2_ for 1 h. The CO_2_ molecules break Ti–C bonds to form Ti–O bonds (Fig. 6g).^82^ The reaction process can be expressed as followed:Ti_3_C_2_ + 3CO_2_ → 3TiO_2_ + 5C

The generated TiO_2_ sheets were anchored on carbon layers, forming the well-preserved 2D-layered architecture. It is worth mentioning that the heating temperature had a great impact on the structure of TiO_2_/C composites. When the heating temperature reached 800 °C, TiO_2_ tended to form particles with lower surface energy instead of sheets, and carbon layers would get thinner because of the oxidation. When the temperature got higher, carbon was completely oxidized so that no carbon layers could be observed under 900 °C calcination.

Moreover, calcination under different atmospheres could also contribute to the formation of different titanium compounds. Guo and co-workers fabricated a 2D-layered C@TiN after one-step nitridation at 750 °C under NH_3_ conditions.^56^ NH_3_ molecules first formed Ti–N bonds by breaking Ti–C bonds, and H atoms provided by NH_3_ reacted with C atoms to generate CH_4_ molecules, which were decomposed into C and H atoms again. These C atoms were deposited on the surface of TiN sheets, contributing to a hybrid C@TiN structure. Huang and co-workers synthesized TiS_2_@NSC nanosheets by annealing under H_2_S/Ar atmosphere from PDA-covered Ti_3_C_2_T_*x*_ precursor, which shed light on the design of effective cathode materials for lithium–sulfur batteries.^98^

### 3.4 Synthesis of 3D nanoflowers

**Fig. 7:**
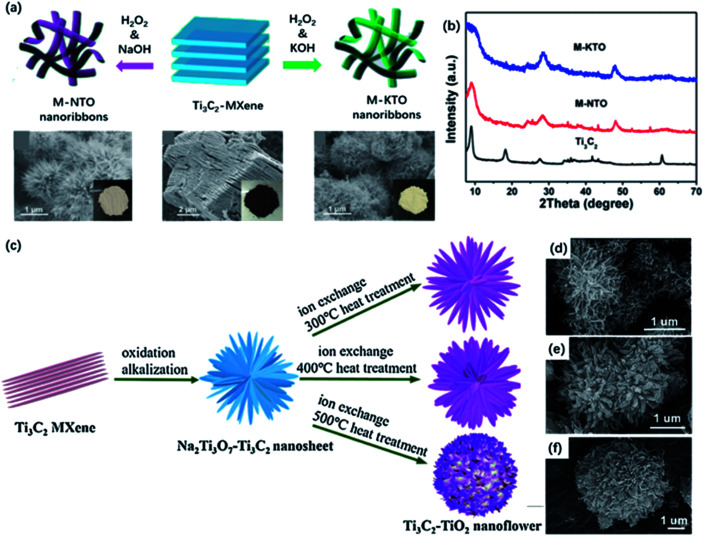
(a) Schematic illustration of the fabrication of M-NTO or M-KTO nanoribbons obtained by hydrothermal treatment in NaOH and KOH, respectively. (b) XRD pattern Ti_3_C_2_, M-NTO and M-KTO.^57^ This figure has been reproduced from ref. 57 with permission from American Chemical Society, copyright 2017. (c) Schematic of the preparation of Ti_3_C_2_/TiO_2_ nanoflowers with different heating temperatures. (d–f) SEM pictures of Ti_3_C_2_/TiO_2_ nanoflowers at different temperatures: (d) 300 °C, 400 °C, 500 °C.^58^ This figure has been reproduced from ref. 58 with permission from Elsevier, copyright 2018.

As mentioned above, MNRs could assemble into a 3D porous framework by stirring in an alkali solution at room temperature, which greatly shortened the ionic diffusion length. Dong and co-workers designed a continuous oxidation and alkalization process to generate MXene nanoflowers *via* hydrothermal treatment in 1 M KOH and NaOH solution with the addition of the small amount of 30 wt% H_2_O_2_ (Fig. 7a).^57^ Compared with MNRs formed in KOH solution, the typical diffraction peak of Ti_3_C_2_ in the XRD pattern completely disappeared. New peaks at 24.3° and 48° corresponding to new species NaTi_1.5_O_8.3_ and K_2_Ti_4_O_9_ were observed (Fig. 7b). In addition, the almost unchanged interlayered space eliminated the possibility of intercalation. The XRD results illustrated that TiO_2_ was oxidized at the early stage, and then the alkalization environment under hydrothermal conditions promoted the formation of sodium or potassium titanates. The as-prepared long curved MNRs were formed and assembled into the urchin-like structure. Up to now, there are many works based on 3D nanoflower structure by hydrothermal treatment of MXene in alkali solution.^99,100^ The unique 3D structure and properties promote the further application in ECS. Furthermore, alkali metal ions could be replaced by H^+^ after immersing in the acid solution for a while and further calcination could generate TiO_2_ nanoflowers. Li and co-workers reported the successful transformation from Ti_3_C_2_ to Ti_3_C_2_/TiO_2_ nanoflowers with improved photocatalysis performance (Fig. 7c).^58^ Ti_3_C_2_/TiO_2_ hybrids were produced after the ion change process in 0.1 M HCl solution and followed by annealing at different temperatures. As the heating temperature increased, the content of anatase TiO_2_ became higher, and the “petals” gradually became wider and shorter according to SEM images (Fig. 7e and f).

Moreover, by controlling the conditions of hydrothermal, the completely oxidized nanoflowers could be obtained instead of 2D nanosheets. Zou and co-workers developed a hierarchical nanoflower-shaped TiO_2_/C composite, achieved by alcohol-thermal decomposition of MXene in a mixing solution of EG, HF solution, and IPA.^59^ It's noteworthy to mention that the modification of solvents to alert the F-containing condition may feasibly control the growth of the lattice plane.^101,102^ The –F groups on the surface make it possible to vary orientational TiO_2_/C hybrids by tuning the growth of crystallographic planes.

## 4 Application

Owing to the controllable morphology and unique properties compared with pristine MXene, MXene derivatives have attracted extensive attention, especially in ECS. In the following section, we outline recent progress based on MXene derivatives with enhanced performance in ECS, mainly including photocatalytic/electrocatalytic hydrogen production, metal ion batteries and lithium–sulfur batteries.

### 4.1 Photocatalytic hydrogen production

The generation of H_2_ from water by photocatalysis is a clean method to convert solar energy into hydrogen fuel. Up to now, a variety of photocatalysts have been developed for the utilization of light-driven hydrogen evolution, such as metal oxides, nitrides, sulfides.^103–106^

However, the single component suffered from the fast photoexcited carrier recombination, resulting in poor H_2_ production properties. In this case, co-catalysts are needed to help capture carriers and prevent the recombination of electrons and holes. Noble metals are the most ideal co-catalysts to promote photogenerated electron transfer and the separation of electrons and holes, but their extremely high price limit their actual applications in photocatalysis. In recent years, MXenes have aroused noteworthy attention in photocatalysis as co-catalysts owing to the excellent electrical conductivity, hydrophilicity, and low Fermi level compared to semiconductors. It has been demonstrated from DFT calculations that the O-terminated Ti_3_C_2_ exhibited near-zero Gibbs free energy for hydrogen adsorption (Δ*G*_H_) and the most positive value of EF, implying high H_2_ evolution activity and ability to capture the photo-induced electrons.^107^

#### 4.1.1 0D quantum dots

**Fig. 8:**
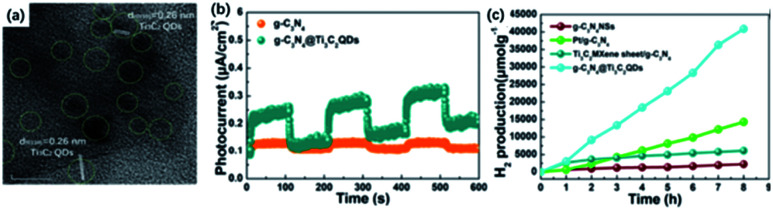
(a) HRTEM images of g-C_3_N_4_@Ti_3_C_2_ QD composites. (b) Photocurrent responses of g-C_3_N_4_@Ti_3_C_2_ QD composites and g-C_3_N_4_. (c) Photocatalytic H_2_ evolution of g-C_3_N_4_@Ti_3_C_2_ QD composites.^42^ This figure has been reproduced from ref. 42 with permission from American Chemical Society, copyright 2019.

Ti_3_C_2_ QDs have drawn noteworthy attention as the co-catalyst for their excellent solvent solubility, attractive edge sites and electronic properties compared with 2D sheets. Moreover, Ti_3_C_2_ QDs could be exited *via* a broad spectrum ranging from visible to NIR, which is also beneficial to facilitate photocatalytic activity. Inspired by the unique properties, Qiao and co-workers applied ultrasound-treated Ti_3_C_2_ NPs as co-catalysts to merge with CdS. Ti_3_C_2_ NPs were symmetrically deposited on the surface of CdS, showing the cauliflower-structured morphology. By adding 2.5 wt% Ti_3_C_2_ NPs, the composites achieved the photocatalytic H_2_ production activity of 14 342 μmol g^−1^ h^−1^, which was 136 times higher than that of pure CdS. The superior photocatalytic activity resulted from the strong combination of two components to facilitate the carrier transfer. Similarly, Li and co-workers designed Ti_3_C_2_ QDs@g-C_3_N_4_ nanosheets *via* a self-assembly method. The as-obtained Ti_3_C_2_ QDs were intimately immobilized on g-C_3_N_4_ nanosheets as confirmed from TEM images (Fig. 8a).^42^ With the loading of Ti_3_C_2_ QDs, the pore-size distribution is relatively dispersed, which would significantly facilitate the charge transfer process. Also, an increased lifetime of charge carriers could be observed on account of the properties of capturing electrons supported by the metallic conductivity of Ti_3_C_2_ QDs. As a result, g-C_3_N_4_@Ti_3_C_2_ QD composite (5.5 wt% Ti_3_C_2_ QDs loaded) showed a greatly-enhanced photocatalytic H_2_ generation activity (5111.8 μmol g^−1^ h^−1^) (Fig. 8b).

#### 4.1.2 2D nanosheets

**Fig. 9:**
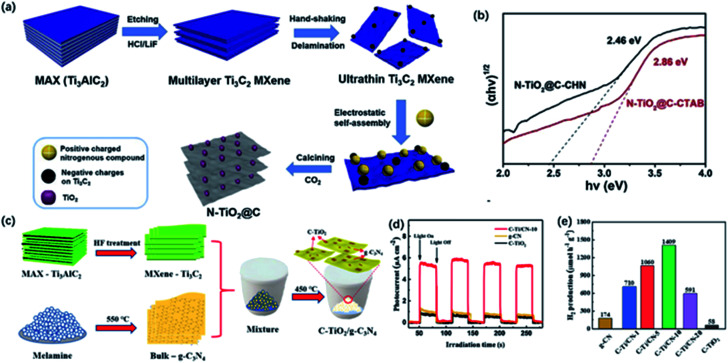
(a) Schematic of the fabrication of the preparation of 2D layered N-TiO_2_@C. (b) The bandgap energy of N-TiO_2_@C.^55^ This figure has been reproduced from ref. 55 with permission from Elsevier, copyright 2019. (c) Schematic illustration of the preparation of C-TiO_2_/g-C_3_N_4_ hybrid. (d) Intermittent transient photocurrent response spectra of C-TiO_2_/g-C_3_N_4_ hybrid. (e) Photocatalytic H_2_ production rate of C-TiO_2_/g-C_3_N_4_ hybrid.^108^ This figure has been reproduced from ref. 108 with permission from Elsevier, copyright 2020.

As we know, TiO_2_ is the most widely studied photocatalyst owing to its high photocatalytic activity, chemical stability, and low cost. However, the limited light absorption and unsatisfactory recombination of photogenerated electron–hole severely reduce its photocatalytic efficiency. In this case, Ti_3_C_2_T_*x*_ MXene served as the titanium resource and carbon skeleton was considered as the proper precursor for the growth of TiO_2_ with enhanced photocatalytic activity. 2D nanosheets show more contact areas, and the intimate interfacial contact between TiO_2_ and nanosheet facilitates the separation of photo-induced carriers and improves the utilization of light.^53^ Wang and co-workers reported the high visible responsive TiO_2_/C composite from 2D Ti_3_C_2_T_*x*_ precursor *via* hydrothermal treatment and followed by annealing at 300, 400 and 500 °C for 0.5, 1, 3, 8 h, respectively.^53^ The TiO_2_/C samples annealing at 400 °C for 1 h showed a significant H_2_ evolution rate (69 μmol g^−1^ h^−1^), which increased nearly 3 times that of the commercial pure P25 under light irradiation. The obvious improvement of photocatalytic activity contributed to the moderate carbon homogeneously distributed on the TiO_2_, which greatly improved the absorption of visible light and facilitated the migration and separation of photogenerated carriers on account of the formed interface of TiO_2_/C. Moreover, by adding element precursors and other materials with high photocatalytic activity, the hybrids exhibited superior performance in H_2_ evolution. Huang and co-workers prepared N-TiO_2_@C nanocomposites with nitrogen-containing cationic compound (CTAB, CHN) modified by calcining under CO_2_ atmosphere (Fig. 9a).^55^ N elements were uniformly dispersed on the surface of the sample thanks to the electrostatic interactions between the positive charged cationic compound and the negatively charged surface of ultrathin MXene sheets. The bandgaps of N-TiO_2_@C-CTAB/CHN samples were estimated to be about 2.86 eV and 2.46 eV, smaller than that of pure TiO_2_ (3.2 eV) (Fig. 9b). Importantly, N-TiO_2_@C showed excellent photocurrent density compared to TiO_2_@C. The doping of N atoms contributed to smaller bandgaps and red-shift light adsorption edge and more efficient photogenerated electrons transmittance according to photoluminescence (PL) spectroscopy, which reduced the recombination rate of photogenerated carriers.

The as-prepared TiO_2_/C could serve as the co-catalysts assisting the transfer of carriers as well. Moreover, g-C_3_N_4_ with layered structure has been proved to be a hopeful candidate for photocatalysis, while co-catalysts are still needed to capture carriers and offer enough active sites to improve overall photocatalysis performance. Han and co-workers prepared C-TiO_2_/g-C_3_N_4_ composite *via* a typical calcining process of pre-synthesized bulk g-C_3_N_4_ from melamine and etched Ti_3_C_2_T_*x*_ at 450 °C for 5 h (Fig. 9c).^108^ After calcination, Ti_3_C_2_ was completely converted to anatase phase, meanwhile, bulk g-C_3_N_4_ was changed to g-C_3_N_4_ sheets, which may enhance the photocatalytic activity of H_2_ generation. C-TiO_2_/g-C_3_N_4_-10 (the ratio of Ti_3_C_2_ to g-C_3_N_4_ is 10) exhibited the highest photocatalytic H_2_ generation rate of 1409 μmol g^−1^ h^−1^, which was about 8 and 24 times higher than g-C_3_N_4_ and C-TiO_2_, respectively (Fig. 9e). Note that the marked hydrogen production activity resulted from the intimate heterojunction between C-TiO_2_ derived from MXene and g-C_3_N_4_, which efficiently facilitate the transfer of photocatalytic carriers and inhibit the recombination of electrons and holes.

#### 4.1.3 3D nanoflowers

In general, 3D nanoflower structures have large specific surfaces, which is convenient for the access of the solvent molecules to the reactive sites easily and shorten the diffusion paths for photoexcited electrons and holes.^109,110^ Li and co-workers synthesized MXene-derived Ti_3_C_2_/TiO_2_ nanoflowers, providing more active sites and shorter diffusion lengths of photogenerated holes and electrons.^58^ In this work, the intimate contact between Ti_3_C_2_ and TiO_2_ and the heterojunction interface generated synergetic effect and Schottky junction, which would effectively inhibit the recombination and bring about more electrons participating in photoreduction for H_2_ evolution.

Compared to 2D sheets, MQDs show excellent solvent solubility and electronic properties, which are beneficial to photocatalytic activity. On the other hand, TiO_2_ derived from Ti_3_C_2_T_*x*_ is the most widely investigated photocatalyst. The intimate interfacial contact between TiO_2_ nanoparticles and 2D sheets could facilitate the separation of photo-induced carriers and optimize photocatalytic activity. More efforts can be made to develop the modification methods of synthesis to better control the beneficial morphology and new hybrids of MXene and MXene derivatives to promote photocatalytic activity.

### 4.2 Electrocatalytic hydrogen evolution reaction

Except for the utilization of solar energy, the conversion of electrical energy to chemical energy like electrocatalytic hydrogen evolution reaction (HER) could be an efficient way of producing H_2_. To date, noble metals and their oxides are considered the most efficient electrocatalysts. However, high cost and scarcity limit their practical applications. In recent years, many noble metal-free catalysts have been developed with high activity in HER, while their poor charge transfer characteristics and limited exposing edges remain problems. MXene with high metallic electrical conductivity and the large surface area attracted huge attention. It has been demonstrated that O-containing groups on the surface are the catalytic active sites that are in favour of the fast charge transfer.^111^ Besides, MXene derivatives with unique morphology have shown enhanced performance in electrocatalytic HER.

#### 4.2.1 1D nanoribbons

**Fig. 10:**
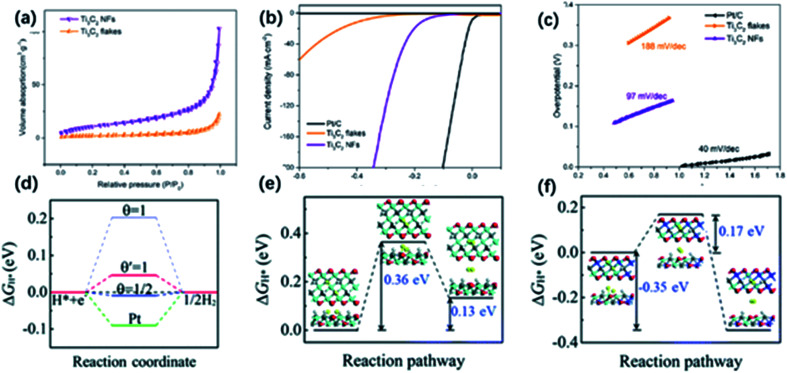
(a) N_2_ absorption–desorption image of Ti_3_C_2_ NFs and Ti_3_C_2_ nanosheets. (b) HER properties of Ti_3_C_2_ NFs: LSV (b) and Tafel slopes (c) of Ti_3_C_2_ NFs, Ti_3_C_2_ nanosheets, and Pt/C electrodes.^51^ This figure has been reproduced from ref. 51 with permission from American Chemical Society, copyright 2018. (d) Δ*G*_H_ value for hydrogen evolution at zero potential (pH = 0) on the edges of Ti_3_C_2_ (blue lines) and MXene nanoribbons (red line) at different H* coverage. (e, f) Δ*G*_H_ value of the Tafel reaction for H_2_ formation on the edges of (e) Ti_3_C_2_ and (f) MXene nanoribbons.^116^ This figure has been reproduced from ref. 116 with permission from Royal Society of Chemistry, copyright 2018.

**Fig. 11:**
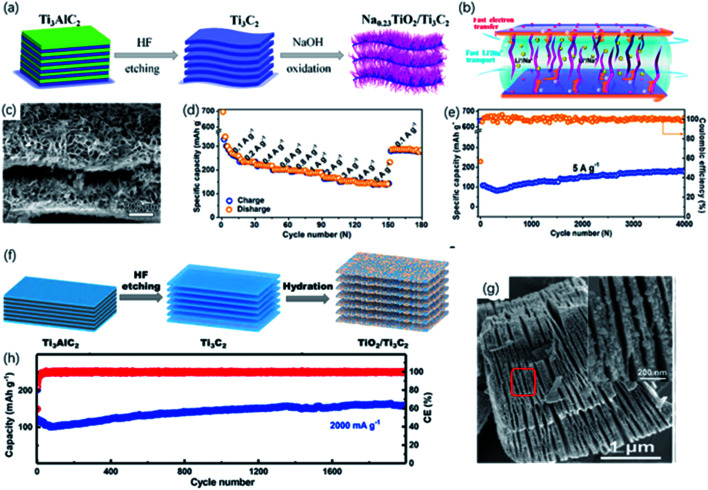
(a) Schematic of the preparation of the sandwich-like Na_0.23_TiO_2_/Ti_3_C_2_ composite *via* alkali treatment. (b) Energy-storage mechanism in Na_0.23_TiO_2_/Ti_3_C_2_ composite. (c) SEM picture of Na_0.23_TiO_2_/Ti_3_C_2_ composite. (d, e) Rate performance (d) and long-term cycling performance at 5 A g^−1^ (e) of Na_0.23_TiO_2_/Ti_3_C_2_ composite.^49^ This figure has been reproduced from ref. 49 with permission from Elsevier, copyright 2018. (f) Schematic diagram of synthesis of the partial-oxidized TiO_2_/Ti_3_C_2_ nanohybrid. (g) FESEM picture of accordion-like TiO_2_/Ti_3_C_2_ nanohybrid. Inset in (g): FESEM of the red zone circled (h) cycling performance of TiO_2_/Ti_3_C_2_ nanohybrid at a 2000 mA g^−1^ (high rate).^117^ This figure has been reproduced from ref. 117 with permission from Elsevier, copyright 2018.

1D nanostructures have shown great potential in HER owing to large specific surface areas and abundant active sites.^112–115^ The structural advantage makes it easy to realize the fast charge-transfer process. Yuan and co-workers developed MXene nanofiber structure with enhancing HER activity because of the high specific surface area and more exposed sites.^51^ Ti_3_C_2_ NFs possessed an increased surface area of 58.5 m^2^ g^−1^ and showed an abundant pore structure based on the N_2_ absorption–desorption isotherm (Fig. 10a). Also, alkali treatment could improve the catalytic performance by increasing the content of oxygen groups on the surface. As a result, the HER polarization curves indicated a lower overpotential (169 mV) at 10 mA cm^−2^ and smaller Tafel slope (97 mV dec^−1^), compared to the Ti_3_C_2_ sheet (overpotential of 385 mV; Tafel slope of 188 mV dec^−1^) (Fig. 10b and c). The impedance spectra of the referencing equivalent circuit suggested much lower charge transfer resistance (*R*_ct_) of Ti_3_C_2_ NFs than that of Ti_3_C_2_ sheets because the 1D structure could shorten the diffusion pathway and promote the charge transfer. The enhanced electrocatalytic activity was also confirmed by first-principles calculations. Yang and co-workers found the edges of the MXene nanoribbons might serve as the reaction sites to adsorb hydrogen species referring to the calculation results.^116^ In particular, Ti_3_C_2_ nanoribbons exhibited great HER activity because of the relatively low adsorption free energy (approaching 0) and small Tafel barrier (0.17 eV), which greatly promoted the charge transfer from metal atoms on the edge to H reactants in the transition state (Fig. 11d–f).

Compared to the MXene sheets, 1D NRs show more exposing active sites and increased surface area, which are beneficial to the HER. In the future, more hybrids of 1D NRs and other electrocatalysts can be produced to shorten the diffusion pathway and promote the charge transfer process.

### 4.3 Metal-ion batteries

In recent years, developing new generation energy storage devices has attracted tremendous attention due to the increasing demand for high energy density and harmless environmental impact. Until now, rechargeable batteries are the most widely used energy storage device, in which the performance mainly depends on the properties of electrode materials. Hence, the development of electrode materials is vital to improving electrochemical performance. Metal-ion batteries featuring high energy capacity, lightweight and long cycle life are the first commercial devices. Since the latest lithium-ion battery was produced in 1991, the development of lithium-ion batteries has attached great importance to the utilization of portable electronic equipment and electric vehicles. MXene showed considerable potential in constructing high-performance electrode material for lithium-ion batteries due to the high conductivity and abundant active sites. As the most studied MXene, Ti_3_C_2_ has a theoretical capacity of ∼320 mA h g^−1^, while it only showed a capacity of ∼120 mA h g^−1^ in the experiments, owing to the influence of the surface termination groups. But high capacities are found in O-terminated MXene, attributed to the formation of bilayer Li atoms between the layers. As confirmed from DFT calculations, Li diffusion barriers are pretty low, resulting in the high rate performance.^121^ Therefore, many strategies, such as oxidation and alkalization, have been developed to optimize the storage of Li-ions.^87,88,99^

#### 4.3.1 1D nanoribbons

The 1D nanostructure is attractive for the storage of metal-ion owing to the short ion diffusion length and little volume change against deformation, which are beneficial for stable and efficient ion storage.^122,123^ Inspired by these excellent properties, the alkalized Ti_3_C_2_ nanoribbons were synthesized and exhibited superior lithium ions storage performance and outstanding long-term cyclability for LIBs, which outperformed most of the reported MXene-based materials.^47,57^ Huang and co-workers studied the partial alkalization of Ti_3_C_2_ nanoribbons for LIBs (Fig. 11a). In ambient alkali conditions, ribbon-like Na_0.23_TiO_2_ was generated on the surface of Ti_3_C_2_ forming the unique sandwich structure (Fig. 11b).^49^ The Na_0.23_TiO_2_/Ti_3_C_2_ delivered a high reversible capacity of 278 mA h g^−1^ at 0.2 A g^−1^ after 400 cycles and exhibited remarkable cycling stability of nearly 100% capacity retention at high rates for up to 400 cycles (Fig. 11c and d). Such improvement was attributed to the efficient ion transfer and steady layered structure without restacking because of the nanoribbon between the layers (Fig. 11e). Compared to lithium-ion batteries, sodium/potassium-ion batteries require suitable ion storage sites considering the radius of ions. The expansion of interlayer distance happened during the first intercalation. Then the pillaring effect of trapped Na^+^ and the swelling effect of penetrated solvent molecules between layers supported the reversible intercalation of Na^+^.^124^ Benefitting from the large porous channel for ion transfer, nanoribbons also behaved well in sodium/potassium-ion batteries. Wu and co-workers synthesized nanoribbons from Ti_3_C_2_ MXene as the anode material for SIBs/PIBs.^57^ The resulting nanoribbon structure exhibited suitable interlayer spaces and macroporosity structure, resulting in an enhanced reversible capacity (191 mA h g^−1^ at 200 mA g^−1^) and long cycling performance.

#### 4.3.2 2D nanosheets

2D MXene derivatives preserve the large panel structure, at the same time, the *in situ* grown TiO_2_ particles contribute to the expanded interlayer space and extra capacity.^118^ Yang and co-workers adopted a facile hydrothermal method to fabricate TiO_2_/Ti_3_C_2_ hybrid, where TiO_2_ nanoparticles were *in situ* formed on Ti_3_C_2_ sheets (Fig. 11f and g).^117^ After oxidation, the interlayer *d*-spacing of hybrids expanded from 9.8 Å to ∼10.2 Å according to XRD results. In the electrochemical tests, the resulting TiO_2_/Ti_3_C_2_ exhibited reversible capacities of ∼267 mA h g^−1^ at a current density of 200 mA g^−1^ and outstanding cyclic stability without apparent capacity decay at the high current rate over 2000 cycles owing to the expanded interlayer spacing for the intercalation and de-intercalation of Li ions (Fig. 11h). Similarly, Li and co-workers developed N-doped Ti_3_C_2_@TiO_2_ composites *via* HNO_3_-assisted etching and further oxidation process.^88^ The N-doped samples showed a higher reversible capacity of 302 mA h g^−1^ at 200 mA g^−1^ after 500 cycles owning to the larger interlayer distance of 12.77 Å.

1D NRs derived from MXene have been comprehensively discussed as the promising electrode of metal-ion batteries these years. However, poor electron conductivity and slow ion transfer limit the capacitance and cycling performance. Future experiments can focus on the combination of MXene derivatives and other energy storage materials with high conductivity to improve the performance of the electrode system.

### 4.4 Lithium–sulfur batteries

Lithium–sulfur batteries have been considered as the promising candidate for next-generation batteries owing to the high theoretical capacity and rich reserves of sulfur. Nevertheless, further practical application is restricted by the poor conductivity of sulfur, huge volume change during cycling, and “shuttle effect” caused by the soluble lithium polysulfides (LiPSs) intermediates with a negative impact on the capacity and cycling performance.^125^ To address these issues, intensive efforts have been made, such as employing conductive carbon-based materials in the system. However, carbon-based materials exhibited poor LiPSs adsorption, especially for high sulfur loading batteries.^126^ In this case, some polar materials, such as transition metal oxides, sulfides, have been inducted as additives. These polar materials exhibited high trapping ability and efficient catalytic effects on the conversion of LiPSs, contributing to the enhanced capacity and cycling performance. Recently, MXene has shown impressive potential as the additives of sulfur hosts owing to the high conductivity, abundant functional groups for the strong interactions with LiPSs.^127–129^ Also, MXene derivatives with different morphology and unique properties showed stronger adsorption and efficient catalyzation, which are beneficial for the capture of LiPSs.

#### 4.4.1 0D quantum dots

**Fig. 12:**
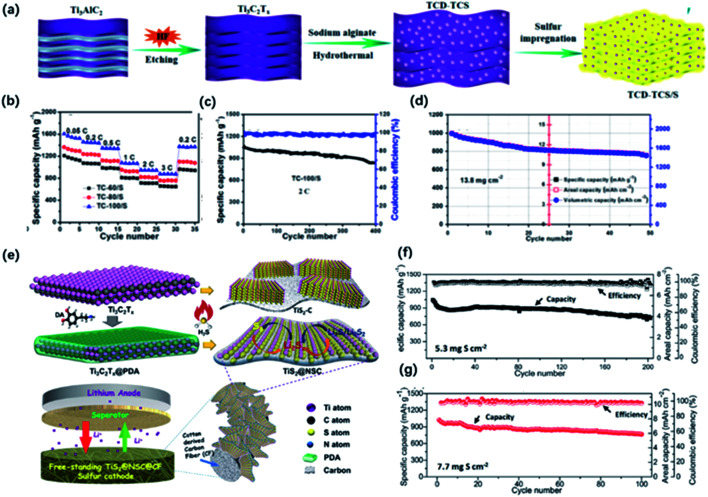
(a) Schematic of the preparation of the hybrid of Ti_3_C_2_ quantum dots and Ti_3_C_2_ sheets (TCD–TCS) and TCD–TCS/S. (b) Rate performance of the TCD–TCS/S tailoring at different temperatures: TC-60/S, TC-80/S, and TC-100/S electrodes with ASL of 1.8 mg cm^−2^. (c, d) Cycling performance of (c) TC-100/S at 2C with ASL of 1.8 mg cm^−2^, (d) 0.05C with high ASL of 13.8 mg cm^−2^.^41^ This figure has been reproduced from ref. 41 with permission from American Chemical Society, copyright 2019. (e) Schematic illustration of the synthesis of sandwich-like, single-layer TiS_2_ nanosheets confined within PDA-derived N, S co-doped porous carbon (TiS_2_@NSC). (f, g) Cycling performance of the freestanding S/TiS_2_@NSC@CFs electrode with different areal sulfur loading at 0.1C.^98^ This figure has been reproduced from ref. 98 with permission from Wiley-VCH, copyright 2019.

The ultrasmall size of 0D quantum dots provides more exposed active sites for the effective trapping of LiPSs in the cathode, which is beneficial for the absorption and localization of polysulfides.^130,131^ Moreover, by taking advantage of the small size and excellent dispersibility, 0D quantum dots could be uniformly dispersed in the system, forming strong chemical interactions with polysulfides even at a high sulfur loading. Xiao and co-workers presented Ti_3_C_2_-derived quantum dots with an average size around 2.5 nm decorated on Ti_3_C_2_ nanosheets *via* hydrothermal treatment (Fig. 12a).^41^ The abundant surface terminations resulting in intimate contact between ultrafine QDs and nanosheets, which was probably attributed to the charge conduction and minimized the irreversible loss of LiPSs when cycling. The hybrid electrode had the theoretical discharge capacity at the sulfur loading of 1.8 mg cm^−2^ and exhibited ultrahigh volumetric capacity (1957 mA h cm^−3^) and areal capacity (13.7 mA h cm^−2^) at a high sulfur loading of 13.8 mg cm^−2^ (Fig. 12b–d).

#### 4.4.2 1D nanoribbons

1D nanoribbon shows a large surface area with many open macropores beneficial to high sulfur loading and fast ionic diffusion. Dong and co-workers designed an all-MXene monolithic electrode for the Li–S battery, where alkalized Ti_3_C_2_ nanoribbons served as the sulfur host, and Ti_3_C_2_ nanosheets as the interlayer on the PP separator.^48^ The formed Ti_3_C_2_ nanoribbons offered abundant macropores and large surface areas for high sulfur loading. Meanwhile, Ti_3_C_2_ nanosheet hindered the shuttle effect of polysulfides. As a result, the fabricated electrode exhibited a reversible capacity of 1062 mA h g^−1^ at 0.2C and the outstanding rate capacity of 288 mA h g^−1^ at 10C.

#### 4.4.3 2D nanosheets

2D layered structure offers a large available surface area for the adsorption of LiPSs and buffering the volume expansion during cycling, while self-restacking still greatly restricts the further utilization of its structural advantage. Through the controlled oxidation, TiO_2_ particles could be generated on the surface of MXene, serving as the adsorption sites to capture LiPSs.^119^ At the same time, the well-preserved 2D plane could guarantee the diffusion of LiPSs in the electrode material. Yang and co-workers synthesized TiO_2_-MXene heterostructures *via* hydrothermal treatment.^120^ The fabricated Li–S battery delivered 800 mA h g^−1^ at 2C and ultralong cycling performance with a high sulfur loading. Similarly, Dong and co-workers designed a hierarchical MXene@TiO_2_ nanoarray structure with abundant mesopores through the controlled solvothermal process, which dramatically facilitates the transport of electrolytes and improves the sulfur loading. Moreover, Huang and co-workers reported a sandwich-like TiS_2_ nanosheet confined by N, S co-doped carbon (TiS_2_@NSC) as a sulfur host after the sulfuration of polydopamine (PDA)-coated Ti_3_C_2_ (Fig. 12e).^98^ The coating of PDA efficiently prevented the restacking of MXene sheets, resulting in the significantly increased specific surface of TiS_2_@NSC (267.3 m^2^ g^−1^) compared to that in TiS_2_@C without PDA coating (4.9 m^2^ g^−1^). TiS_2_@NSC delivered a discharge capacity of 920 mA h g^−1^ at 0.2C after 120 cycles and maintaining 695 mA h g^−1^ at 1C after 200 cycles (Fig. 12f and g). Later in the prolonged cycles, TiS_2_@NSC showed a lower overpotential, which suggested the efficient catalyzation of solid–liquid and liquid–liquid conversion.

Different dimensions of MXene derivatives show great potential as the electrode in Li–S battery, providing more adsorption sites to capture LiPSs. The porous structure also guarantees the efficient diffusion of LiPSs in the electrode, even at high sulfur loading. In the future, considerate attention can be devoted to the combination of nanomaterials for the design of electrodes and modification of separators.

## 5 Conclusions

2D transition metal carbide/nitrides, especially the Ti_3_C_2_T_*x*_ derivatives, have been considered promising materials for ECS. Herein, we summarize the typical synthetic routes of MXene and four different forms of derivatives, 0D quantum dots, 1D nanoribbons, 2D nanosheets and 3D nanoflowers. Furthermore, recent works involving their applications in ECS are presented. The advantages and expected applications of MXene derivatives are summarized in Table 3.

**Table 3:** Advantages and expected applications of MXene derivatives

Structure of MXene derivatives	Advantages	Expected applications
0D MQDs	Extraordinarily small size, good solubility	Photocatalysis
1D MNRs	Shorter transport length, more exposed active sites	Electrocatalysis, metal ion batteries
2D MNSs	Perfect base material with large planes, good electron conductivity	Photocatalysis, rechargeable batteries
3D MNFs	Porous structure with large surface area, structural stability	Photocatalysis, rechargeable batteries

It should be pointed out that synthetic methods determine the morphology and chemical properties of the derivatives. In particular, 0D MQDs exhibit extraordinarily small size and luminescence properties because of the quantum confinement and edge effects. The unique characteristics of MQDs have shown great potential in many fields, including sensing, biomedical, catalysis and energy storage. 1D nanoribbons and 3D nanoflowers, which are obtained *via* shaking and hydrothermal treatment under alkali conditions, respectively, form a porous network structure. The as-obtained titanates with 3D porous structures have shown infinite potential in energy storage and conversion applications. Moreover, the partial or complete oxidation of MXene sheets produces TiO_2_ with a well-preserved layered structure. The *in situ* formed interface optimizes the charge transfer, realizing the further application of TiO_2_ in photocatalysis.

By taking advantage of structural superiority and intrinsic properties, MXene derivatives showed improved performance in ECS. Although enormous progress has been achieved on the structural design and applications of MXene derivatives, there are still some remaining challenges. For example, proper attention should be devoted to deeply understand the forming mechanism of nanostructure to better control the beneficial morphology of MXene derivatives. Figuring out simple and efficient synthesis routes that make the size and morphology of resulting products (0D, 1D, 2D, 3D) controllable to improve their performance in ECS applications. Developing new material systems for enhanced performance based on the matching of physical mechanics and corresponding applications. Research related to the nanostructure derived from MXene is still in an early stage of development, so more effort should be paid into the design of MXene derivatives in the future.

## Conflicts of interest

There are no conflicts to declare.

## Supplementary Material
